# Cardiovascular disease biomarkers derived from circulating cell-free DNA methylation

**DOI:** 10.1093/nargab/lqad061

**Published:** 2023-06-28

**Authors:** Rafael R C Cuadrat, Adelheid Kratzer, Hector Giral Arnal, Anja C Rathgeber, Katarzyna Wreczycka, Alexander Blume, Irem B Gündüz, Veronika Ebenal, Tiina Mauno, Brendan Osberg, Minoo Moobed, Johannes Hartung, Kai Jakobs, Claudio Seppelt, Denitsa Meteva, Arash Haghikia, David M Leistner, Ulf Landmesser, Altuna Akalin

**Affiliations:** Bioinformatics & Omics Data Science Platform, Berlin Institute for Medical Systems Biology, Max-Delbrück-Center for Molecular Medicine Berlin, Berlin, Germany; Charité - Universitätsmedizin Berlin, corporate member of Freie Universität Berlin, Humboldt-Universität zu Berlin, and Berlin Institute of Health, Department of Cardiology, Campus Benjamin Franklin, Berlin, Germany; DZHK (German Centre for Cardiovascular Research), partner site Berlin, Germany; Charité - Universitätsmedizin Berlin, corporate member of Freie Universität Berlin, Humboldt-Universität zu Berlin, and Berlin Institute of Health, Department of Cardiology, Campus Benjamin Franklin, Berlin, Germany; DZHK (German Centre for Cardiovascular Research), partner site Berlin, Germany; Bioinformatics & Omics Data Science Platform, Berlin Institute for Medical Systems Biology, Max-Delbrück-Center for Molecular Medicine Berlin, Berlin, Germany; Berlin Institute of Health (BIH), Berlin, Germany; Bioinformatics & Omics Data Science Platform, Berlin Institute for Medical Systems Biology, Max-Delbrück-Center for Molecular Medicine Berlin, Berlin, Germany; Bioinformatics & Omics Data Science Platform, Berlin Institute for Medical Systems Biology, Max-Delbrück-Center for Molecular Medicine Berlin, Berlin, Germany; Bioinformatics & Omics Data Science Platform, Berlin Institute for Medical Systems Biology, Max-Delbrück-Center for Molecular Medicine Berlin, Berlin, Germany; Bioinformatics & Omics Data Science Platform, Berlin Institute for Medical Systems Biology, Max-Delbrück-Center for Molecular Medicine Berlin, Berlin, Germany; Bioinformatics & Omics Data Science Platform, Berlin Institute for Medical Systems Biology, Max-Delbrück-Center for Molecular Medicine Berlin, Berlin, Germany; Bioinformatics & Omics Data Science Platform, Berlin Institute for Medical Systems Biology, Max-Delbrück-Center for Molecular Medicine Berlin, Berlin, Germany; Charité - Universitätsmedizin Berlin, corporate member of Freie Universität Berlin, Humboldt-Universität zu Berlin, and Berlin Institute of Health, Department of Cardiology, Campus Benjamin Franklin, Berlin, Germany; DZHK (German Centre for Cardiovascular Research), partner site Berlin, Germany; Charité - Universitätsmedizin Berlin, corporate member of Freie Universität Berlin, Humboldt-Universität zu Berlin, and Berlin Institute of Health, Department of Cardiology, Campus Benjamin Franklin, Berlin, Germany; DZHK (German Centre for Cardiovascular Research), partner site Berlin, Germany; Charité - Universitätsmedizin Berlin, corporate member of Freie Universität Berlin, Humboldt-Universität zu Berlin, and Berlin Institute of Health, Department of Cardiology, Campus Benjamin Franklin, Berlin, Germany; DZHK (German Centre for Cardiovascular Research), partner site Berlin, Germany; Charité - Universitätsmedizin Berlin, corporate member of Freie Universität Berlin, Humboldt-Universität zu Berlin, and Berlin Institute of Health, Department of Cardiology, Campus Benjamin Franklin, Berlin, Germany; DZHK (German Centre for Cardiovascular Research), partner site Berlin, Germany; Universitätsklinikum Frankfurt am Main, Medizinische Klinik 3, Klinik für Kardiologie und Angiologie, Germany; Charité - Universitätsmedizin Berlin, corporate member of Freie Universität Berlin, Humboldt-Universität zu Berlin, and Berlin Institute of Health, Department of Cardiology, Campus Benjamin Franklin, Berlin, Germany; DZHK (German Centre for Cardiovascular Research), partner site Berlin, Germany; Charité - Universitätsmedizin Berlin, corporate member of Freie Universität Berlin, Humboldt-Universität zu Berlin, and Berlin Institute of Health, Department of Cardiology, Campus Benjamin Franklin, Berlin, Germany; DZHK (German Centre for Cardiovascular Research), partner site Berlin, Germany; Berlin Institute of Health (BIH), Berlin, Germany; Charité - Universitätsmedizin Berlin, corporate member of Freie Universität Berlin, Humboldt-Universität zu Berlin, and Berlin Institute of Health, Department of Cardiology, Campus Benjamin Franklin, Berlin, Germany; DZHK (German Centre for Cardiovascular Research), partner site Berlin, Germany; Universitätsklinikum Frankfurt am Main, Medizinische Klinik 3, Klinik für Kardiologie und Angiologie, Germany; Charité - Universitätsmedizin Berlin, corporate member of Freie Universität Berlin, Humboldt-Universität zu Berlin, and Berlin Institute of Health, Department of Cardiology, Campus Benjamin Franklin, Berlin, Germany; DZHK (German Centre for Cardiovascular Research), partner site Berlin, Germany; Berlin Institute of Health (BIH), Berlin, Germany; Bioinformatics & Omics Data Science Platform, Berlin Institute for Medical Systems Biology, Max-Delbrück-Center for Molecular Medicine Berlin, Berlin, Germany

## Abstract

Acute coronary syndrome (ACS) remains a major cause of worldwide mortality. The syndrome occurs when blood flow to the heart muscle is decreased or blocked, causing muscle tissues to die or malfunction. There are three main types of ACS: Non-ST-elevation myocardial infarction, ST-elevation myocardial infarction, and unstable angina. The treatment depends on the type of ACS, and this is decided by a combination of clinical findings, such as electrocardiogram and plasma biomarkers. Circulating cell-free DNA (ccfDNA) is proposed as an additional marker for ACS since the damaged tissues can release DNA to the bloodstream. We used ccfDNA methylation profiles for differentiating between the ACS types and provided computational tools to repeat similar analysis for other diseases. We leveraged cell type specificity of DNA methylation to deconvolute the ccfDNA cell types of origin and to find methylation-based biomarkers that stratify patients. We identified hundreds of methylation markers associated with ACS types and validated them in an independent cohort. Many such markers were associated with genes involved in cardiovascular conditions and inflammation. ccfDNA methylation showed promise as a non-invasive diagnostic for acute coronary events. These methods are not limited to acute events, and may be used for chronic cardiovascular diseases as well.

## INTRODUCTION

Despite a marked mortality reduction driven by improved diagnosis and medical care, ischemic heart disease, a.k.a. acute coronary syndromes (ACS) remains a leading health burden in the 21st century (1). There is an utmost demand for novel diagnostic tools that refine stratification and monitoring of patient care to improve therapy choice. Chest discomfort is the predominant initial symptom of ACS, but a combination of criteria is required to achieve the diagnosis of acute myocardial infarction. First, patients with persistent electrocardiogram (ECG) abnormalities are defined as ST-segment elevation myocardial infarction (STEMI) patients (2). STEMI is a life-threatening episode accounting for around 30% of ACS cases that can lead to ventricular fibrillation or sudden cardiac arrest and requires immediate intervention (3). Plasma biomarkers such as cardiac troponin I (TnI) and T (TnT), or creatine kinase isoenzyme MB (CK-MB) determine the extent of myocardial injury and are additionally used for diagnosis of non-ST-segment elevation myocardial infarction (NSTEMI) (4–7). Recent advances in cardiac-specific-troponin (cTn) assays such as high sensitive cardiac Troponin (hs-cTn) have increased their sensitivity and diagnostic value (8). Although this biomarker is highly sensitive, cardiomyocyte-specific and allows estimation of cell stress injury of only cardiac origin, the effects of severe hypoxia on other cell types present in the ischemic area remain unknown. Patients with normal ECG and no rise of myocardial injury markers (hs-cTn) and chest pain at rest are defined as unstable angina (UA). UA patients have a lower risk of death and may benefit from less invasive strategies within 72 h (9) of symptom onset. The causes of UA can be numerous and independent of a thrombotic event, but patients often get the same invasive intervention as NSTEMI. It is currently unknown how much the diagnosis of UA predicts future myocardial infarction, but it is widely anticipated to be a potential warning indication. Respective markers could help here in the treatment and prevention setting of acute myocardial infarctions. It would also be extremely beneficial to identify new non-invasive biomarkers that could improve the stratification between thrombotic (Type I MI) or non-thrombotic events (Type II MI) leading to different types of myocardial infarction (10). More accurate and less invasive diagnostic methods are needed. CcfDNA recently emerged as a new type of biomarker that is associated with many diseases. Nucleosome-sized DNA fragments released from dying apoptotic or necrotic cells make up the ccfDNA, which circulate for a short time in body fluids before they are cleared mainly by the liver (11,12). Increased concentrations of ccfDNA have been detected in many conditions: several types of cancer (13–15), acute and chronic systemic inflammations (16), sepsis (17), stroke (18) and myocardial infarction (19), arising as potential biomarkers for many pathologies. Recently, several studies demonstrated a strong correlation between ccfDNA and cardiovascular disease risk factors and status (19–21). For example, levels of ccfDNA have been associated with severity in AMI patients (20) as well as with cardiometabolic risk factors (19–21). However, a whole genome methylation by bisulfite sequencing of ccfDNA has not been utilized so far for stratification of ACS patients. The only approach in this respect was undertaken by focusing on the methylome of human heart chambers, identifying a cluster of cytosines alongside the FAM101A locus. This cardiac-specific methylated region adjacent to the gene FAM101A has been investigated as a possible biomarker for human cardiomyocyte death (19,22). One of those studies found significant differences in the methylation of the six CpGs in this region on ccfDNA from STEMI patients and sepsis (19). More commonly, analyzing ccfDNA fragments by next-generation sequencing (NGS) is used for the detection of different types of cancer signatures by identifying mutations related to cancer types (15), monitoring transplant rejection by detecting increased levels of ccfDNA from organ donor (23) and fetal genetic diseases screening (24). Besides variant detection, DNA methylation patterns of ccfDNA enable the quantification of cell death in a tissue specific manner, leading to a better understanding of the pathophysiological processes and can serve as biomarkers for diseases (25,26).

In this work, we used whole genome bisulfite sequencing (WGBS) of patient-derived ccfDNA and computational analysis to associate methylation patterns to ACS types: STEMI, NSTEMI and UA. We observed changes in cell type proportions associated with the disease status using methylation pattern deconvolution. We obtained differentially methylated regions (DMR) from ACS patients compared to healthy controls, as potential biomarkers for ACS patient stratification. We also developed a more cost-effective targeted sequencing approach to validate the potential biomarkers in an independent cohort of patients. Furthermore, we developed an R package and reproducible notebooks for other researchers to investigate ccfDNA methylation as potential biomarkers for other diseases. In summary, this work shows the utility of ccfDNA methylation markers in cardiovascular disease diagnostics and serves as a blueprint for expansion of ccfDNA methylation markers into other cardiovascular disease areas.

## MATERIALS AND METHODS

### Ethics approval and consent to participate

The study has been conducted according to the declaration of Helsinki and was approved by the Berlin State Ethics Committee in Berlin, Germany (EA4/122/14 and EA1/270/16).

### Patient recruitment and sample collection

A total of 29 individuals were recruited for the first discovery cohort. This group included 8 healthy individuals (control), 8 STEMI patients, 7 NSTEMI patients and 6 UA patients, with an average age of 61.5 ranging from 38 to 84. Twenty-eight individuals were men and one healthy control was a woman. For our validation, using a target sequencing approach, we used a cohort of 2 healthy subjects, 4 STEMI, 3 NSTEMI and 2 UA patients (Table S1).

All included patients gave their consent after being fully informed about the study and its purpose.

Fresh blood samples from patients and healthy controls were collected into Na-Citrate tubes (BD), centrifuged at 1200g at room temperature (RT) for 10 min in a swing-bucket centrifuge. The supernatant (SN) was transferred to a 15 ml Falcon tube (Greiner) and the tube was spun again at 1200g for 10 min. Double-centrifuged plasma was then transferred into a screw cap plasma collection tube (3 ml Kisker) and immediately frozen at -80°C until ccfDNA extraction. STEMI patients were immediately submitted to PCI after confirmed ST-elevation via ECG and parallel troponin measurement.NSTEMI are patients that show no ST elevation in the ECG and were tested for hsTroponin T using two-time points and showed either a 15–20% troponin rise or a level >52 ng/ml and were submitted to the catheter lab for PCI within a maximum of 72 h. UA patients do not show a rise in troponin and have a largely normal troponin base level, but suffer from chest pain at rest and were also submitted for PCI below 72 h of admission. All subjects gave their written consent after being fully informed about the study and its purpose before the blood was drawn. Patient characteristics, including age, disease status, concentration and the total amount of ccfDNA obtained, and cardiac biomarkers are described in Table S1.

### Measurement of clinical parameters

All clinical parameters and biomarkers were measured directly in plasma or serum by Labor Berlin using standardized assays and procedures routinely used in diagnostics for Charite hospital.

### ACS classification by classical biomarkers

To classify disease severity based on classical biomarkers, multinomial logistic regression models are used via the multinom () function in *nnet* package. We have created single variable multinomial logistic regression models to predict ACS type: STEMI, NSTEMI and UA. In each model, we used one of the ccfDNA levels in blood or classical clinical biomarkers as the predictor variable. Patients which did not have data available for certain markers (NA values) were excluded from the corresponding model. The misclassification rate is calculated as the percentage of misclassified samples output by the model.

### ccfDNA extraction

ccfDNA was extracted from freshly thawed double centrifuged (1200g at RT) citrate-plasma using the Qiagen QIAamp Circulating Nucleic Acid Kit (Cat. #55114) and collected using ultrapure nuclease-free water in a total volume of 50 μl and quantified using a Qubit fluorometer 2.0 and the Qubit dsDNA HS Assay kit (Cat. # Q32854). Fragment size was confirmed and validated using Agilent Tape Station 2200 and the ccfDNA screentape or the hsD1000 screentape. Total amount of cfDNA was then normalized to the amount of plasma it was extracted from.

### Whole-genome bisulfite sequencing (WGBS)

Isolated ccfDNA was sent to Novogene and rechecked for quality and concentration by the company. Bisulfite conversion and sequencing was performed using their low-input BS-seq (PBAT) protocol. Data was analyzed using previously in-house programmed R software packages (27).

### Targeted methylation sequencing

Input amount and pulldown were optimized using sheared genomic DNA (ZymoResearch Cat. # D5014). A total of 10 ng of isolated cell-free DNA per sample, quantified and checked for quality, was used as starting material. For enzymatic conversion as an alternative to bisulfite conversion, we applied the NEBNext Enzymatic Methyl-seq Module (Cat. #E7120S) together with the Nonacus Cell3TMTarget: Library Preparation kit. Up to 8 libraries of individually adapter-tagged samples were pooled to a total amount of 1 μg for probe hybridization and capture enrichment. Captured library DNA was quantified and quality checked using Qubit Fluorometer and qPCR as well Agilent 2200 TapeStation with High Sensitivity D1000 reagents and screentape. Samples were loaded onto a SP flow cell and sequenced using the Novaseq 6000 platform acquiring 400 million reads.

### Sequencing quality control, alignment to the reference and methylation calling

Raw reads were processed using the PiGx BSseq pipeline (28). First, they were checked for quality with FastQC (,version 0.11.8). Then, the read sequences were trimmed using Trim Galore wrapper (29) (version 0.6.4, cutadapt version 2.6, with arguments ‘–clip_R2 19 –three_prime_clip_R2 30’ ‘–clip_R1 9’ ‘–three_prime_clip_R1 30’), removing Illumina adaptors and sequences with quality Phred score smaller than 20. For the WGBS samples (discovery), bwa-meth (version 0.2.2) (30) was used to map reads to reference the human genome (hg38), Picard MarkDuplicates (https://broadinstitute.github.io/picard/,version 2.20.4) for deduplication and methylDackel software package (https://github.com/dpryan79/MethylDackel, version 0.5.1, with arguments –minDepth 1 -q 5 -p 5) for methylation calling. For the targeted sequencing (validation), the reads were aligned using Bismark (version 0.20.1, with arguments -N 0 -L 20 –pbat), deduplicated using samblaster (version 0.1.24) and methylKit (version 1.16.0) (27) was used for methylation calling.

### Cell type/tissue deconvolution

The deconvR R package was created for the omics-based deconvolution of cfDNA to cell types of origin. The deconvolute function within deconvR (https://github.com/BIMSBbioinfo/deconvR) offers four choices of models, non-negative least squares (NNLS, nnls package v.1.4), support vector regression (SVR, e1071 package v.1.7.4), quadratic programming (QP, quadprog package v.1.5.8), and robust linear regression (RLM, MASS package). The best model for methylation-based deconvolution was determined as follows. Using the simulateCellMix from the deconvR package, we simulated a dataset containing 1000 mixed samples based on the reference atlas from Moss *et al.* (26) with duplicate CpGs removed (25 tissues/cell types and 6105 CpGs). This simulated dataset was deconvoluted using all four different models and the predictions were compared to the actual cell type proportions, to calculate the accuracies and weaknesses of each model. The lowest root-mean-square error (RMSE) value was obtained with the NNLS model, followed by the RLM model.

The comprehensive array-based human cell type methylation atlas as generated by Moss *et al.* (26) was extended by adding three more heart tissues to the full reference methylation atlas (25 tissues/cell types and ∼390K CpGs) and performing tissue-specific CpG feature selection as follows. EPIC Methylation-array data for the right atrium auricular region (*n* = 2, ENCSR517JQA and ENCSR280LMY), heart left ventricle (*n* = 2, ENCSR515ZCU and ENCSR190PQG) and the coronary artery (*n* = 2, ENCSR688OHW and ENCSR582BMR) were acquired from the ENCODE portal (31). The methylation-array idat files were renamed to respective red/green channels and then pre-processed using the script provided via the meth_atlas GitHub repository (https://github.com/nloyfer/meth_atlas) to normalize the data using an arbitrary reference sample, filter by *P*-value, sex chromosomes and bead number, and finally remove SNPs and non-CpG sites. The pre-processed ENCODE methylation data was merged with the full reference atlas based on CpG probe ID to construct the raw extended methylation atlas (28 tissues/cell types and ∼390K CpGs). Then CpGs with missing values or sites where the row-wise variance was <0.1% were omitted and replicates were pooled per tissue.

For the selection of tissue-specific CpGs from our extended atlas two approaches were followed. First, the top 100 most hypomethylated and hypermethylated CpGs for each cell type were selected as described by Moss *et al.* (26) with the minor adjustment of defining a list of already used CpGs in any tissue, to select another CpG for a tissue if the top hits are already in use. Briefly, the methylation atlas was scaled by dividing each row of the atlas by the summed methylation values of the respective row, then for each cell type, the top 100 hypermethylated CpGs with the highest scaled methylation values were selected and recorded to prevent repeated selection of the same CpGs. This procedure was repeated for the reversed scaled methylation matrix to identify the top 100 hypomethylated CpGs per cell type. Secondly, using the unscaled extended atlas, the dmpFinder function of the minfi R package (32) was used to identify for each cell type the 200 most differential CpGs compared to any other tissue.

The sets of tissue-specific CpGs were joined and based on those, neighboring CpGs within a distance of 50 bp and pairwise-specific CpGs were added as explained in Moss *et al.* (26)

To deconvolve the WGBS ccfDNA samples into cell types of origin, genomic loci were first mapped to CpG probe IDs using the BSmeth2Probe function in deconvR. The genomic locations of CpG probe IDs were specified according to the Illumina Infinium MethylationEPIC v1.0 B5 Manifest File. The cell type of origin proportions per sample were estimated using the deconvolute function of deconvR employing the NNLS model and the extended cell type specific CpG methylation signature matrix (28 Cell types) as reference.

### Differential methylation

The differential methylation was called on tiled regions (500 bp sliding windows with a step size equal to 500 bp) using the R package methylKit (27) (version 1.16.0), *q*-value cutoff 0.01 via SLIM method (33) and minimal methylation difference of 25%.

### DMR annotation

The DMRs were annotated by Genomic Regions Enrichment of Annotations Tool (GREAT) (34) using the rGREAT package (release 3.12). The model used was the ‘Basal plus extension’, annotating genes on proximal regions (5 kb upstream, 1 kilobase downstream) plus distal (up to 1000 kb). We also annotate the DMRs for CpG regions, genomic regions and regulatory regions using the AnnotateR package (35) and additionally we annotate enhancer regions using the chromHMM bed files from the Roadmap Epigenomics Project (36) (Core 15-state model).

The list of gene symbols obtained was submitted to DisGeNET (v 7.0), a comprehensive platform integrating information on human disease-associated genes and variants (37), using its R package disgenet2r (v.0.0.9, database = all). The gene symbols obtained from GREAT were converted to Entrez gene ids and used for DisGeNET enrichment analysis (enrichDGN) with R package DOSE (v 3.12) with parameters *P*-value cutoff <0.05, adjusted *P*-value <0.2, minGSSize = 2, maxGSSize = 500, pAdjustMethdod = BH, background genes = whole human genome). We looked for heart-related disease by searching terms from the disease name and disease class name from DisGeNET, assigning to each DMR the annotation obtained.

### Linear models of the methylation levels on DMRs

In order to obtain ACS type associated DMRs, we defined the ACS type as a numeric vector of severity (UA = 1, NSTEMI = 2, STEMI = 3). This follows the classical clinical understanding of ACS where UA is the least severe and STEMI is the most severe type of ACS. We have used linear regression models using R *lm ()* function, we have constructed following models, where *Y* is either disease severity as explained above or the clinical biomarkers:


$$\begin{equation*}Y\ \sim\ {\beta }_0\ + {\beta }_1 \cdot \ DNAmeth + {\beta }_2 \cdot ccfDNAleve{l}_{}\end{equation*}$$

We have constructed such a model for each DMR. This way we associated DMRs with disease severity by testing regression coefficients by t-test, and retained DMRs that are significantly associated with disease severity. Similarly, we have also associated DMRs with clinical markers simply building models for each clinical marker where the response variable Y is the clinical marker. In addition, in order to test if DNA methylation levels provide important value for prediction of disease severity over ccfDNA level we have also built a reduced model for each DMR where only ccfDNA levels are used as a predictor variable. We compared these reduced models with the full models using ANOVA test. This way we would be able to control for ccfDNA levels and their contribution to model fit.

### DMR validation analysis with targeted sequencing

After the detection of a systematic bias of methylation percentages in the targeted sequencing data (see Supplementary Figure S5), log transformation using pseudo-counts (log((observed + 1)/( (100 – observed) + 1)) and quantile normalization using the R package's ‘preprocessCore’ (version 1.58.0) function ‘normalize.quantiles.use.target ()’ were applied. In addition, ComBat adjustment from the R package ‘sva’ (version 3.44.0) using the function ‘ComBat ()’ with subsequent back transformation, served to normalize the data. Mean differences (condition - healthy control percentage of methylation) were computed per DMR and compared to the discovery cohort's values. The Pearson correlation coefficient was computed using the R package's ‘ggpubr’ (version 0.4.0) function ‘stat_cor ()’. The PCA on DMRs which were associated with disease groups was generated using the R ‘stats’ (version 4.2.1) ‘prcomp ()’ function.

### Machine learning models for prediction of ACS type

In order to predict the ACS type from ccfDNA methylation profiles, we trained three predictive models (random forest (RF), partial least squares regression (PLS), penalized multinomial regression (PMR)) on a 70/30 train-test-split of the discovery data. We have used *PLS* and *randomForests* packages via the *caret* package to build and test the models. We used the methylation values from 193 DMRs shared between discovery and validation as features. The features were pre-processed by centering and scaling. We have used Cross-Validation (10 fold, repeated 10 times) for model assessment. We then applied these models on the batch normalized validation cohort.

## RESULTS

### ccfDNA levels and classical clinical biomarkers are predictive of ACS status but can not accurately differentiate ACS types

For diagnosis and patient characterization, we measured classical biomarker levels of the study participants (Table S1) next to ECG and the patients were assigned to the ACS groups by a clinician according to ESC guidelines (Collet J. P., ESC guidelines, EHJ2021). High-sensitive cardiac TroponinT (hs-cTnT) was mostly undetected in healthy controls and had low levels in UA patients, and highest values in STEMI and NSTEMI (Supplementary Figure S1A). As expected, the left ventricular ejection fraction (LVEF) was higher in healthy controls (ranging from 64% to 74%) and lower in ACS patients, with some patients from the UA group showing very low values (minimal value of 30%, Supplementary figure S1B). For creatine kinases (CK) we see an increase of the levels with increasing severity of ACS (STEMI exhibits the highest median, followed by NSTEMI and UA medians) (Supplementary Figure S1C–F).

**Figure 1.**
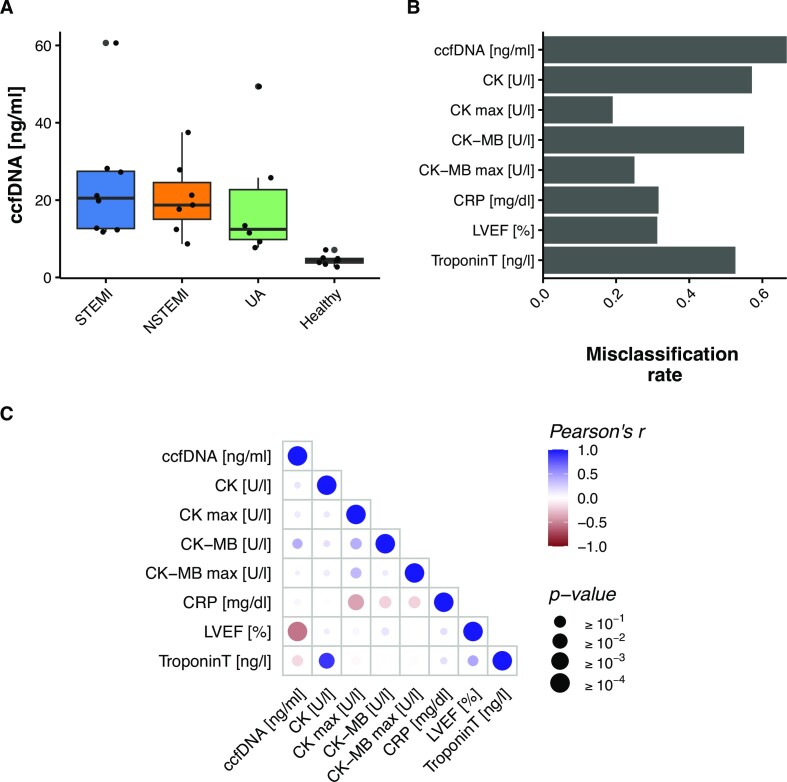
(**A**) Total amount of extracted ccfDNA normalized to plasma volume. (**B**) Misclassification of ACS type by classical biomarkers and ccfDNA levels. (**C**) Correlation analysis of the classic cardiac biomarkers and ccfDNA concentration. LVEF, Left ventricular ejection fraction. CRP, C reactive protein. Troponin T, troponin T high-sensitive. CK, creatinine kinase. CK max, maximum value of CK measured. CK-MB, creatinine kinase isoform MB. CK-MB max, maximum value of CK-MB measured.

The total amount of ccfDNA extracted per mL of plasma from the 29 discovery samples ranged from 2.68 ng (healthy control) to 60.65 ng (STEMI) (Figure 1A, Table S1). All ACS groups showed higher levels of ccfDNA when compared with the healthy control group (Wilcoxon rank-sum test *P*-values 0.0001 for STEMI, 0.0003 for NSTEMI and 0.0006 for UA), confirming previous findings where ccfDNA levels were raised on diseased states. However, total ccfDNA was not statistically different between ACS types, preventing the distinction of ACS types solely by ccfDNA levels. This finding shows that ccfDNA quantity by itself cannot be a very specific marker for ACS stratification, which is also evident when we tried to classify patient samples for the ACS type using ccfDNA levels in a multinomial logistic regression (Figure 1B). In addition, using clinical biomarkers in the same fashion as predictor variables to multinomial logistic regression models also do not yield highly predictive models to distinguish ACS types (misclassification rate is between 20% and 60% for different models with different predictor variables, Figure 1B).

In addition, we checked the Pearson correlation between all the classic biomarkers and the concentration of ccfDNA (for ACS patients only). The classical biomarkers are an important part of the diagnosis although ccfDNA concentration in plasma was inversely correlated with left ventricular ejection fraction (LVEF) (*R*^2^ = 0.56) and unexpectedly, weakly inversely correlated to troponin. On the other hand, ccfDNA was weakly positively correlated to CK and CK-max and positively correlated to CK-MB (*R*^2^ = 0.42) (Figure 1C). These findings show that traditional biomarkers and ccfDNA levels have a complex relationship. Although ccfDNA levels show correlation to multiple classical markers, just the ccfDNA level cannot account for the variation observed in other markers in this cohort of patients.

### Cell type proportions obtained via deconvolution of ccfDNA methylation are associated with ACS

**Figure 2.**
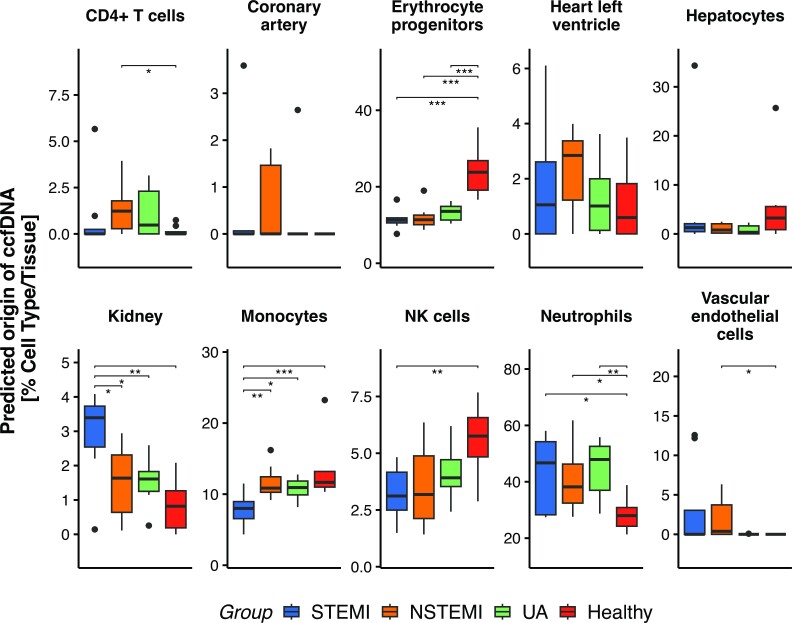
Predicted cell type/tissue proportions per ACS group. Using a comprehensive methylation atlas and non-negative least squares method, the putative origin of ccfDNA could be deconvoluted. (Only cell/tissue types of interest are shown).

Since ACS causes reduced blood flow to the heart and is associated with inflammatory processes, we expect that the cell types that give rise to ccfDNA might be disease specific. We used methylation-based cell type deconvolution to quantify the cell type composition that gave rise to patient-derived ccfDNA. To this end, we applied WGBS on ccfDNA samples obtained from the ACS patients and healthy controls. Our quality control analysis showed that we had a satisfactory bisulfite (BS) conversion rate, ranging from 97.92% to 99.63% and the average CpG read coverage ranged from 4.2 sequenced reads to 9.1 (Supplementary Figure S2). Briefly, our deconvolution method leverages cell type specific methylation patterns in the shape of a signature matrix. The deconvolution algorithm makes use of those patterns to find optimal proportions of cell types that might give rise to the observed bulk DNA methylation pattern. We used a cell type specific CpG methylation signature matrix built from a comprehensive methylation atlas (26) extended with additional heart tissue samples (see Methods for details). The deconvolution algorithm outputs the percentage of cell cell types observed in plasma rather than ccfDNA amount attributed to cell types (the ccfDNA per cell type can be found in Supplementary Figure S6 and Table S3). Percentage of cell types in plasma provides a normalized quantity that is not influenced by ccfDNA amount per patient which is variable but uniformly high compared to healthy samples. Overall, ccfDNA associated with blood cells was the most abundant in all samples, with neutrophils presenting the most abundant cells, ranging from 21% (in the healthy control sample) to 61% (in NSTEMI sample). Previously, granulocytes were found to be the most abundant blood cells in healthy donors (average 32% of the cell composition) (26), matching our results for neutrophils, the most abundant type of granulocytes, with an average of 28% for the healthy group. In our data, in all ACS groups, neutrophils were elevated when compared with the healthy control group. The neutrophils are known to infiltrate infarcted areas in the first hours after ischemia and they play a major role in the subsequent acute inflammation (38). We also could detect increased CD4^+^ T cells (in NSTEMI and UA). On the other hand, ccfDNA from monocytes was decreased in STEMI and proportions of natural killer (NK) cells, erythrocyte progenitors and hepatocytes were reduced in all ACS types (Figure 2). We also note that increased ccfDNA is associated with kidney tissue in all ACS types and specifically in STEMI patients. Acute kidney injury is known to be associated with ACS (39). Furthermore, compared to healthy donors, ACS patients showed an increase in vascular endothelial cells, heart left ventricle and coronary arteries in ACS type specific manner (Figure 2), which indicates there might be ACS type specific tissue damage specific for respective ACS types.

The decoration indicates Wilcoxon test *P*-values between boxplots (significance levels *P-value < 0.05, **P-value < 0.01, ***P-value < 0.001).

### Identifying differentially methylated regions as biomarkers specific to ACS type

**Figure 3.**
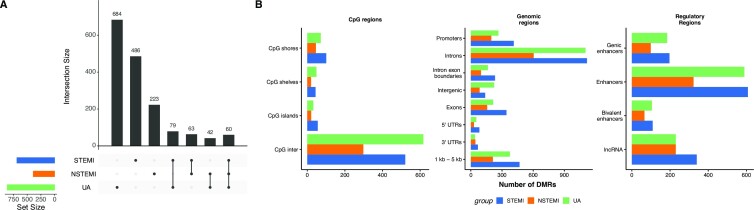
(**A**) DMRs shared across the three ACS groups (compared to healthy control). (**B**) CpG regions, genomic and regulatory annotations for DMRs.

In order to further investigate disease-specific ccfDNA methylation markers, we conducted a tiled differential methylation analysis using logistic regression based statistical testing as implemented in the *methylkit* R package. We compared ACS types to healthy subjects in a pairwise manner. Using a threshold of minimal 25% difference on methylation between the ACS type and healthy subjects and a q-value maximum of 0.01, we identified a total of 688 DMRs in STEMI patients, 388 in NSTEMI patients and 865 in UA. Of those, 486 were STEMI specific, 223 were NSTEMI specific and 684 UA specific. Figure 3A illustrates how the DMRs are shared across the three groups. Those disease specific DMRs are relevant as candidate biomarkers to classify patients in ACS types.

Using all identified DMRs, we generated a principal component analysis (PCA). The results show a clear separation of healthy patients from the ACS groups (Supplementary Figure S3). The UA group is also separated from STEMI and NSTEMI, while the latter two groups are not clearly separated from each other. However, the ability to differentiate UA and healthy patients from NSTEMI and STEMI is already of great use, as the ECG is used as a first stratification of STEMI and NSTEMI. In addition, further filtering of DMRs to strictly disease specific biomarkers are possible as we have shown in the following sections.

Next, we wanted to check if genomics features were associated with the obtained DMRs and if they were associated with disease related genes. For this purpose, we assigned genes that are likely to be regulated by those DMRs using the regulatory-region association tool GREAT (34). Supplementary Figure S4 shows the number of genes per region for each group, and also the distance from DMRs to the transcription start sites (TSS). Additionally, we annotated DMRs with CpG island-associated features, genomic features (such as intron/exon, UTRs etc.), Epigenomics Roadmap enhancers and long non-coding RNAs (lncRNA). Most DMRs were in the open sea, out of CpG islands (CpG inter) (564 for STEMI, 329 for NSTEMI and 765 for UA) (Figure 3B). The genomic annotations show most DMRs on introns (around 60% of all DMRs, Figure 3B) and a large part of it is annotated as enhancers (608 for STEMI, 323 for NSTEMI and 589 for UA) and lncRNAs (340 for STEMI, 230 for NSTEMI and 230 for UA) (Figure 3 B). This points out that differences are possibly driven by cell type specific regulatory regions.

**Figure 4.**
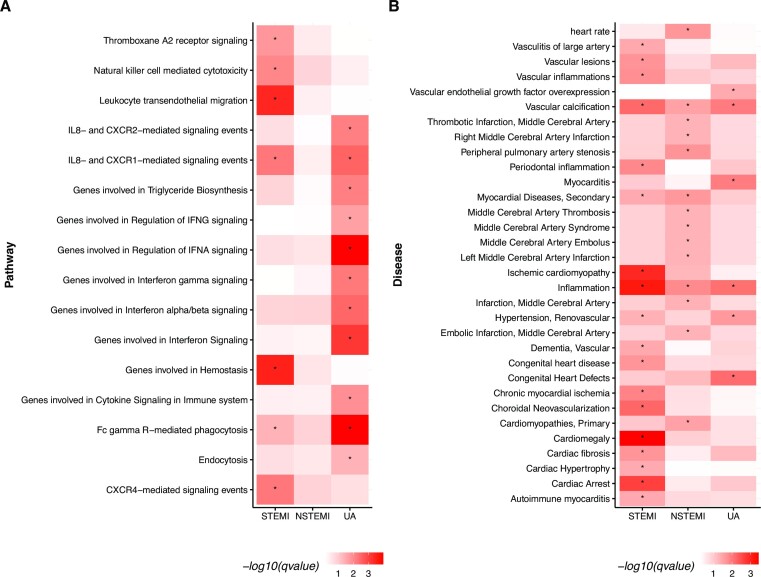
(**A**) GREAT gene set enrichment for DMRs. Enriched canonical pathways from MSigDB CP (curated gene sets), with adjusted *P*-values <0.05 in at least one group are shown. The colors represent the adjusted *P*-values (–log_10_ based). (**B**) DisGeNET enrichment analysis showing cardiovascular/inflammatory diseases with adjusted *P*-values <0.05 in at least one group. The colors represent the adjusted *P*-values (–log_10_ based). The stars (*) indicate *P*-value >0.05.

Following this, we also performed a gene set enrichment test (using the curated canonical binomial test) for the genes associated with DMRs. Here we found pathways involved in the regulation of immune response, hemostasis and phagocytosis enriched for the three ACS groups (Figure 4A). For STEMI, the most significantly enriched pathway was ‘Genes involved in hemostasis’ followed by ‘Leukocyte transendothelial migration’ (adjusted *P*-values 0.0003 and 0.0004, respectively). The hemostasis gene set contained genes involved in coagulation, a process known to be triggered during ACS events (40). For NSTEMI, we could not detect enriched pathways using the conservative threshold adjusted p-value of 0.05. The lowest adjusted p-value was 0.12 (unadjusted p-value 0.003, Regulation of p38-alpha and p38-beta). For UA, the most enriched pathways were ‘Genes involved in Regulation of IFNA signaling’ and ‘Fc gamma R-mediated phagocytosis’ (both adjusted p-values 0.0001). Fcγ receptors (FcγR) are plasma membrane-associated receptors for IgG and the pentraxins C-reactive protein (CRP), a known risk factor for cardiovascular diseases, and its role on inflammation in cardiovascular disorders have been already investigated (41).

Using the DisGeNET database for gene-disease associations (37), we assigned the DMR-related genes to diseases. In total, 74.13% of DMRs from STEMI, 72.68% from NSTEMI and 61.62% from UA were annotated with heart-related diseases. The high proportion of DMRs regulating genes involved in heart-related diseases provided us with additional evidence that those DMRs are promising biomarker candidates. Later, we ran a DisGeNET enrichment analysis to check if the DMRs were statistically enriched (*q*-value < 0.05) for ACS related diseases. We found 505 diseases enriched for STEMI, 253 for NSTEMI and 326 for UA. Filtering by cardiovascular and inflammatory-related diseases, 18 diseases were enriched for STEMI and the most significant was ‘Cardiomegaly’ (*q*-value 0.00038). For NSTEMI, 14 cardiovascular/inflammatory diseases were enriched, with ‘Inflammation’ being the most enriched (*q*-value 0.01). For UA, only six cardiovascular/inflammatory diseases were enriched with ‘Congenital Heart Defects’ presenting the most enriched (*q*-value 0.007). Figure 4B shows all the cardiovascular/inflammatory diseases enriched in at least one group, with an adjusted *P*-value cutoff of 0.05.

### DMRs are associated with ACS types independently of ccfDNA levels in plasma

**Figure 5.**
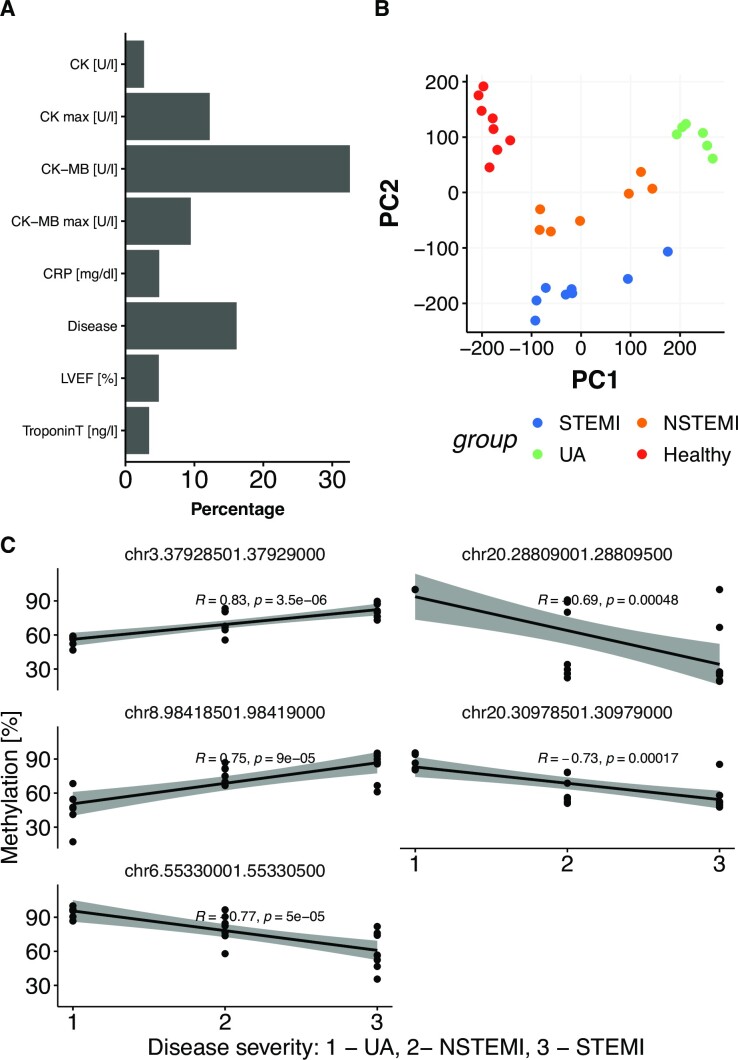
(**A**) Percentage of the discovery DMRs showing significant association with biomarkers and disease group on linear model adjusted by ccfDNA concentration on plasma. (**B**) PCA on methylation levels of 254 DMRs found significantly associated (*P* < 0.05) with ACS types encoded as disease severity using linear models. (**C**) Correlation plot with the top five DMRs most significantly associated with disease groups.

Since the classic clinical biomarkers cannot be effectively used to stratify ACS patients in this cohort (Figure 1B & Supplementary Figure S1), we decided to further investigate the potential of using DMRs for stratification. While the unique 1637 discovered DMRs were all potential markers for separating each ACS group from healthy individuals (because the differential methylation analysis was done in comparison to the control), they are not necessarily useful for discriminating ACS types. Therefore, we decided to further narrow down DMRs to a subset that are strongly associated with the disease. For this purpose, we used linear models predicting numeric disease severity encoding of ACS types or clinical biomarkers from methylation values of DMRs across samples using ccfDNA amount as a covariate (see methods). The disease severity encoding simply shows UA as the least severe and STEMI as most severe which is generally accepted (42). We have fit such models for each DMR to distinguish DMRs that are associated with disease and clinical biomarkers. We found 254 (15.51%) DMRs significantly associated (*P*-value ≤ 0.05) with disease severity, showing that those DMRs were good candidates for disease stratification The details of those DMRs can be found in Table S4 and S5). From those, 158 DMRs were annotated as involved in cardiovascular diseases or inflammation using DisGeNET (37) and 163 overlapped with enhancers from the Roadmap epigenomics project (36). Figure 5A shows the percentage of DMRs significantly associated with clinical biomarkers and ACS type (shown as ‘disease’) encoded as disease severity. Throughout this analysis we controlled for ccfDNA levels in the linear model by adding it as a covariate. As expected, in almost all the linear models ccfDNA levels did not have a significant association with ACS types encoded as disease severity.

Following this, we performed a PCA on the methylation levels of the 254 DMRs which were significantly associated with the disease group (Figure 5B). We observed that this narrower list of DMRs achieved complete separation of ACS types and healthy samples. In Figure 5C, we can see the top five DMRs which are most significantly associated with disease severity. These analyses suggest that DMRs can be used as biomarkers that can distinguish ACS versus healthy samples as well as different ACS types.

### Disease-specific DMRs are validated on an independent patient cohort

In order to validate the discriminative DMRs, we developed a targeted sequencing approach and applied it on an independent cohort of patients. We used 2 healthy subjects, 4 STEMI, 3 NSTEMI and 2 UA patients as the validation cohort. The participants' characteristics can be found in Table S1. Overall, we targeted 18831 CpGs that are either involved in disease-specific DMRs or part of the deconvolution signature matrix, and 75% of those targeted CpGs were covered with at least 5 reads for each sample.

We ran a differential methylation analysis with the validation samples restricting it to the regions of discovery cohort DMRs (using a q-value cutoff < 0.01). We found that 570 of 615 DMRs for STEMI, 308 of 338 for NSTEMI and 252 of 636 for UA were also differentially methylated on the validation samples (DMR subset characterisation in Supplementary Table S2). When also taking directionality of methylation difference into account to ensure that hypo- or hypermethylation of the validation cohort compared to the controls was present with the same effect as in the discovery cohort, the numbers decreased to 566, 306 and 248 DMRs respectively. However, the effect size (methylation difference) is in general lower in the validation cohort.

As from the 1637 unique discovery cohort DMRs only 1314 unique validation DMRs were available due to sequencing coverage, percentages in the following refer to this subset.

**Figure 6.**
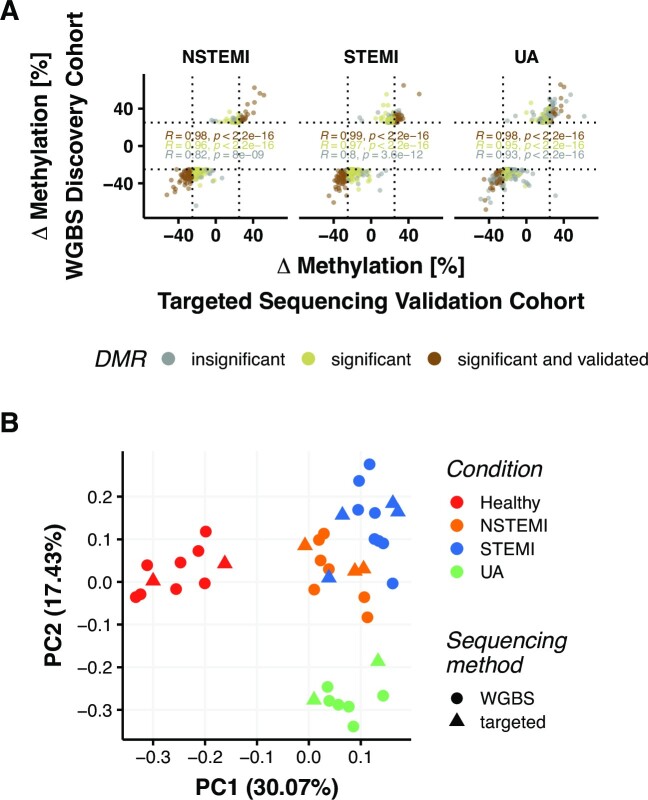
DMR validation in validation cohort. (**A**) DMRs from DM analysis compared to healthy controls (*n* = 1589). Using a 25% methylation difference cutoff 37.3% (115 out of 308 DMRs) of NSTEMI, 30.0% (171 out of 570) of STEMI and 32.5% (82 out of 252 DMRs) of UA DMRs from the discovery cohort could be validated in the validation cohort. Linear Pearson correlation coefficients are stated and dotted lines indicate absolute 25% cut offs. (**B**) PCA on DMRs found significantly associated (*P* < 0.05) with disease groups on linear models in discovery cohort (*n* = 193). Clustering of samples according to condition without regard to the sequencing method.

After applying an absolute 25% cutoff to differential methylation and by keeping the *q*-value cutoff <0.01, 171 STEMI, 115 NSTEMI and 82 UA DMRs remained significant. Quantile normalization and ComBat adjustment of methylation percentages ensured comparability between sequencing techniques and showed the validation of 23% unique DMRs for the validation cohort. These DMRs satisfy q-value and the stringent methylation difference cutoffs in the validation cohort as well (Figure 6A). However, a larger proportion of discovery cohort DMRs (STEMI: 92%; NSTEMI: 91%; UA: 39%) are significantly differentially methylated in the validation cohort when the stringent methylation cutoff is not applied and only the *q*-value cutoff is considered (Figure 6A). In addition, a strong linear correlation of methylation difference values between discovery and validation cohorts (NSTEMI: *R* = 0.98, *P* < 2.2e–16; STEMI: *R* = 0.99, *P* < 2.2e–16; UA: *R* = 0.98, *P* < 2.2e–16) was evident. When using the 254 DMRs from the discovery cohort which were identified to distinguish best between ACS types, we could again observe good separation of the different ACS types and healthy subjects via PCA (Figure 6B) regardless of the different sequencing techniques used in validation and discovery cohorts. Only one STEMI sample from the validation cohort was evident to present as an outlier. In addition, we built multivariate predictive models using multiple machine learning methods (random forests: RF, penalized multinomial regression: PMR and partial least squares regression: PLS, see methods for details). In all cases, the models were trained on the discovery cohort and tested on the validation cohort. We have achieved almost perfect accuracy across models (RF: 0.8182, PLS: 1, PMR: 1, see also Supplementary Figure S7 for cross-validation results). The loss in accuracy in the RF model is due to a single STEMI sample that clusters with the NSTEMI group as can already be seen in Figure 6B.

## DISCUSSION

In recent years, ccfDNA emerged as an interesting new molecular biomarker for diverse diseases (12,16–18,24). However, most of the studies investigated the increase in the total amount of ccfDNA, and the correlation of ccfDNA levels with the severity of the diseases. While useful, such an approach is limited because it cannot discern which tissue is being damaged, nor can it measure the level of injury. The ccfDNA fragments contain DNA methylation patterns which enable their unique assignment to the cell type where the fragment originated. ccfDNA methylation profiling has been used to determine the cellular contributors of the damaged tissue, for example, in islet transplantation and sepsis (26).

In the present study, we present a proof of principle approach, by using WGBS to *de novo* investigate differential methylation profiles of ccfDNA in three different ACS types and a healthy control group. We analyzed both the ccfDNA methylation-based cell composition and the changes of methylation in response to the disease state. Additionally, we used a validation cohort to validate the discovered DMRs, using a targeted sequencing approach. The targeted sequencing approach greatly reduces the profiling costs, increases the speed and makes the method amenable for possible clinical usage.

As previously shown, we found that all ACS groups had increased levels of ccfDNA, when compared to the healthy control group (Figure 1). Interestingly, both MI groups showed slightly higher amounts of ccfDNA than UA. The difference was however not statistically significant, possibly due to the lack of statistical power. A different study reported similar, linearly increasing levels of ccfDNA from UA, NSTEMI and STEMI (43). It is important to state that the ccfDNA fragments can originate from different cell types, and not only cardiomyocytes.

In fact, our cell composition analysis showed an increased proportion of neutrophils in ACS, a cell type known to be involved in the immune response to MI (38). Neutrophils are the first cell type recruited to the site of injury and contribute different functions in cardiovascular diseases (44). During ACS, neutrophils release extracellular traps. Neutrophil extracellular traps (NETs) are secreted structures formed by decondensed chromatin, histones and neutrophil granular proteins, which have been proposed to contribute to ccfDNA (45). These structures have pro-thrombotic activity and their increased levels are related to larger infarct size and major cardiovascular events (44,46).

On the other hand, we observed a decrease in the proportions of erythrocyte progenitors in the ACS groups. Contrary to the other cell types, ccfDNA from those cells does not reflect cell death but rather the process of erythrocyte maturation, when the progenitors lose their nuclei (26,47). The decreased proportion of erythrocyte progenitors might reflect a shift in the hematopoietic process, increasing the production/maturation of immune cells while decreasing erythrocyte maturation. Simultaneously, we also observed an increase of CD4^+^ T-cells, especially in NSTEMI and UA. Cumulative evidence from animal models showed a double role of CD4^+^ T-cells in ischemia-reperfusion injury and tissue recovery (48). We did not detect ccfDNA from the heart's left and right atriums in any of the ACS groups. These results are consistent with the rarity of the atrial infarction, due to the lower oxygen demand of the atrium, when compared to the ventricle. We detected on average 1% of cells from the heart left ventricle in healthy controls, with small increases in ACS, where NSTEMI was on average the most prominent (however the maximum value was observed in a STEMI patient).

Our differential methylation analysis found a total of 1637 DMRs when comparing each of the ACS groups with the healthy controls (Figure 3). Those DMRs clearly separated the ACS groups from healthy controls. These DMRs are mostly located on introns and enhancer regions. The genes they are associated with are clearly associated with coronary artery disease and inflammation (See Figure 4B). The pathways associated with the same genes indicate immune system related pathways such as ‘Leukocyte Migration’ and ‘IFN signaling ’ (Figure 4A). In addition, one of the most significantly enriched pathways for the STEMI DMR set was ‘Genes involved in hemostasis’. The hemostasis gene set contained genes involved in coagulation, a process known to be triggered during ACS events. In summary, our DMR set can be associated with disease relevant genes and processes.

However, the initial DMR set lacked the power to accurately separate the STEMI from NSTEMI patients (Supplementary Figure S3). In order to distill the initial DMR set, we have employed linear modeling to find DMRs where the methylation levels are significantly changed between the different disease groups. This procedure resulted in a set of 254 DMRs, and significantly improved the disease patient stratification (Figure 5B). The reduction in the number of DMRs which are necessary for accurate patient stratification, reduces the sample preparation costs and implies a possible clinical application. Interestingly, 96 of those DMRs are currently not known to be involved in cardiometabolic diseases or inflammation.

In order to validate our findings, we used a targeted sequencing approach. We managed to cover ∼75% of the targeted regions, which was expected due to the technical limitations of probe design. We are able to validate ∼23% unique DMRs using stringent cutoffs. While investigating the DMRs, we observed that the majority of ACS related DMRs are located in intronic regions. The intronic DNA methylation events were also previously associated with the etiology of dilated cardiomyopathy (49) and coronary artery disease (50).

It is of utmost importance to mention that the methylation pattern of the released ccfDNA goes beyond the cell death of cardiomyocytes. It highlights that multiple cell types contribute to ccfDNA, through different biological processes in a disease like acute coronary syndrome. Through the ccfDNA methylation signatures, we are measuring information about a whole spectrum of cellular functions related to acute heart disease, with the final goal of improving both the patient stratification and long-term outcome prediction. Usage of ccfDNA methylation as biomarkers is, however, not limited to acute events, but could be used for measuring disease progression in chronic conditions, such as coronary artery events and heart failure. This utility merits the expansion of ccfDNA methylation biomarkers into larger clinical investigation efforts for cardiovascular diseases.

## CONCLUSIONS

Here, we show the potential of using a set of ccfDNA DMRs for ACS patients’ stratification without undermining the reliability in identifying of STEMI and NSTEMI via ST elevation through ECG and the standardized guidelines of the gold standard of hsTnT measurements. By using 254 DMRs we were able to separate the groups without any other cardiac biomarker without needing any additional clinical finding or biomarker. We were able to apply a targeted sequencing approach using our identified DMRs in order to validate our findings in the second cohort of patients in a more cost-effective way. We also publish an R package and reproducible code base to carry out similar analysis for different diseases. In the future, we plan to expand the cohort types and sizes and to improve the targeted sequencing method to make it useful as a non-invasive tool in clinical settings.

## DATA AVAILABILITY

For reasons of reproducibility scripts are provided on https://github.com/BIMSBbioinfo/ccfDNA_ACSS_manuscript with a comprehensive Readme.md file on the scripts’ content (permanent DOI: https://doi.org/10.5281/zenodo.8009561). Links for public access to methylKit objects (tabix format) for discovery and validation cohort are provided at the end of the Readme.md file on the github repository linked above. Sequence data has been deposited at the European Genome-phenome Archive (EGA), which is hosted by the EBI and the CRG, under accession number EGAS00001007263.

## Supplementary Material

lqad061_Supplemental_Files

## Notes

Present address: Brendan Osberg, Max-Planck-Institut für molekulare Genetik, Berlin, Germany.
