# Supplementary material for: Cardiovascular disease biomarkers derived from circulating cell-free DNA methylation
Source: NAR Genom Bioinform. 2023 Jun 28;5(2):lqad061. doi: 10.1093/nargab/lqad061 (PMC10304763; doi:10.1093/nargab/lqad061)
Supplement: lqad061_Supplemental_Files [file lqad061_supplemental_files.zip › SupplementaryFigures.pdf]

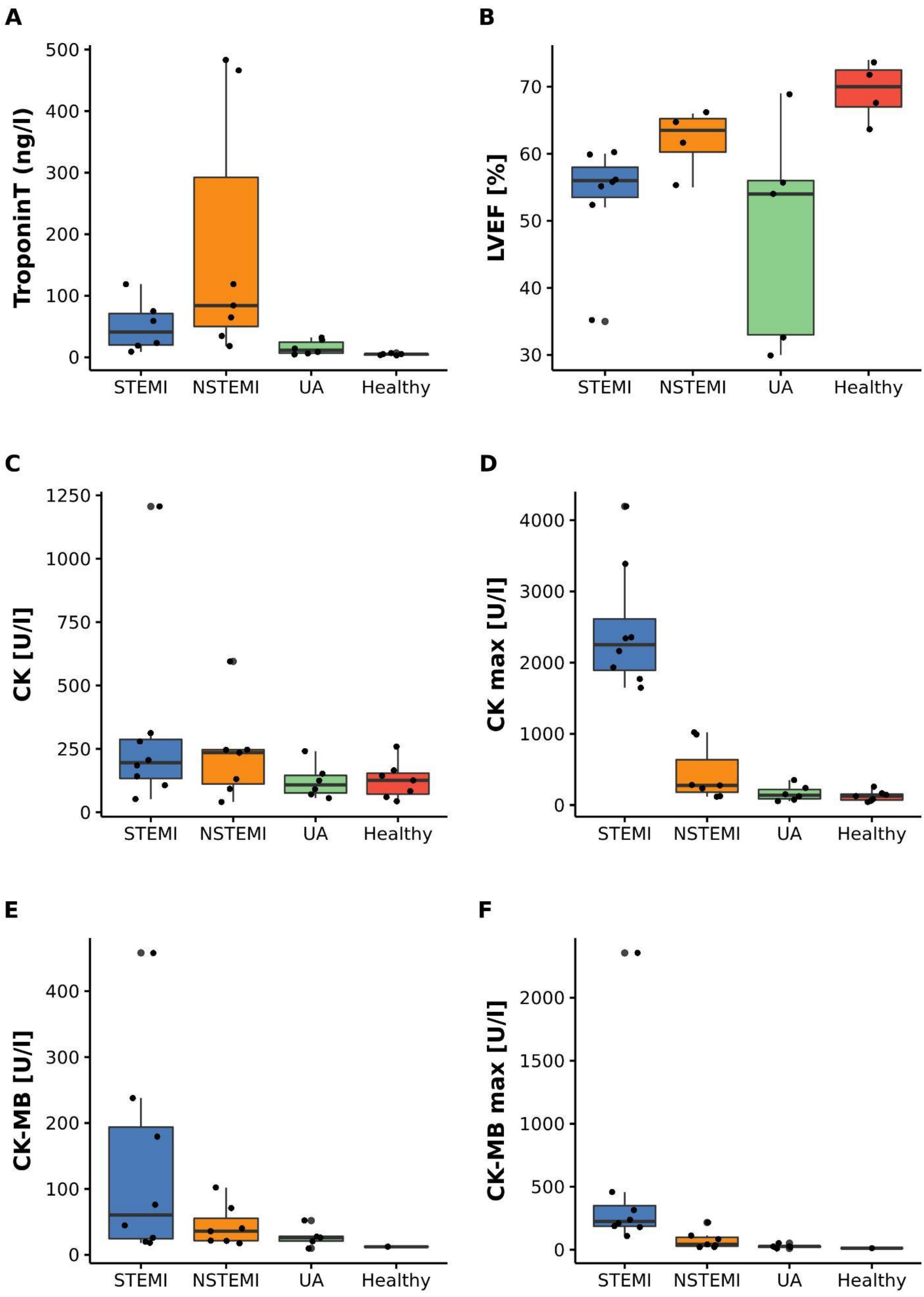

Figure S1: Biomarker values for each patient group representing an ACS type or healthy subjects.

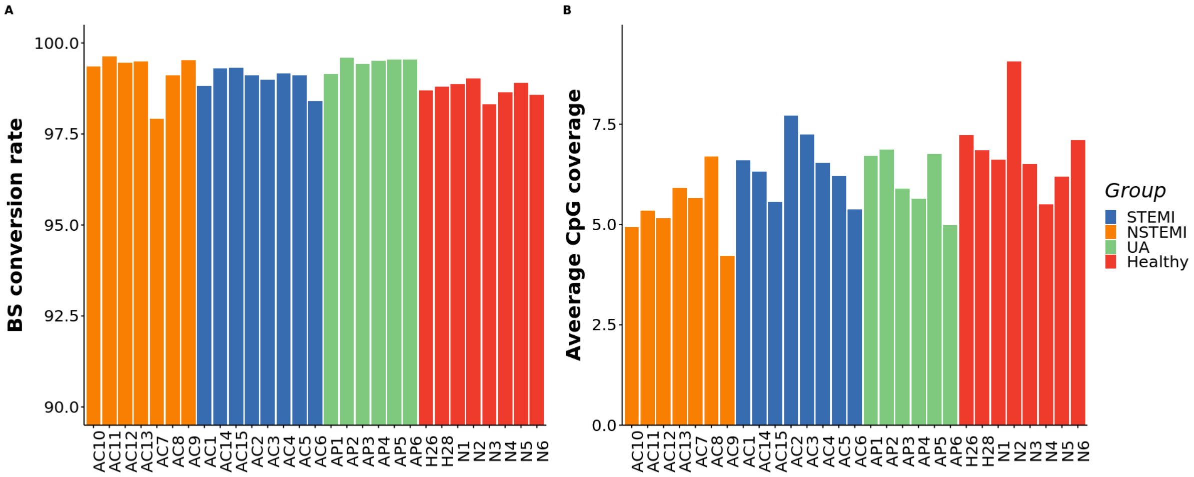

Figure S2: A - BS conversion rate. B - Average CpG coverage for the cfDNA samples.

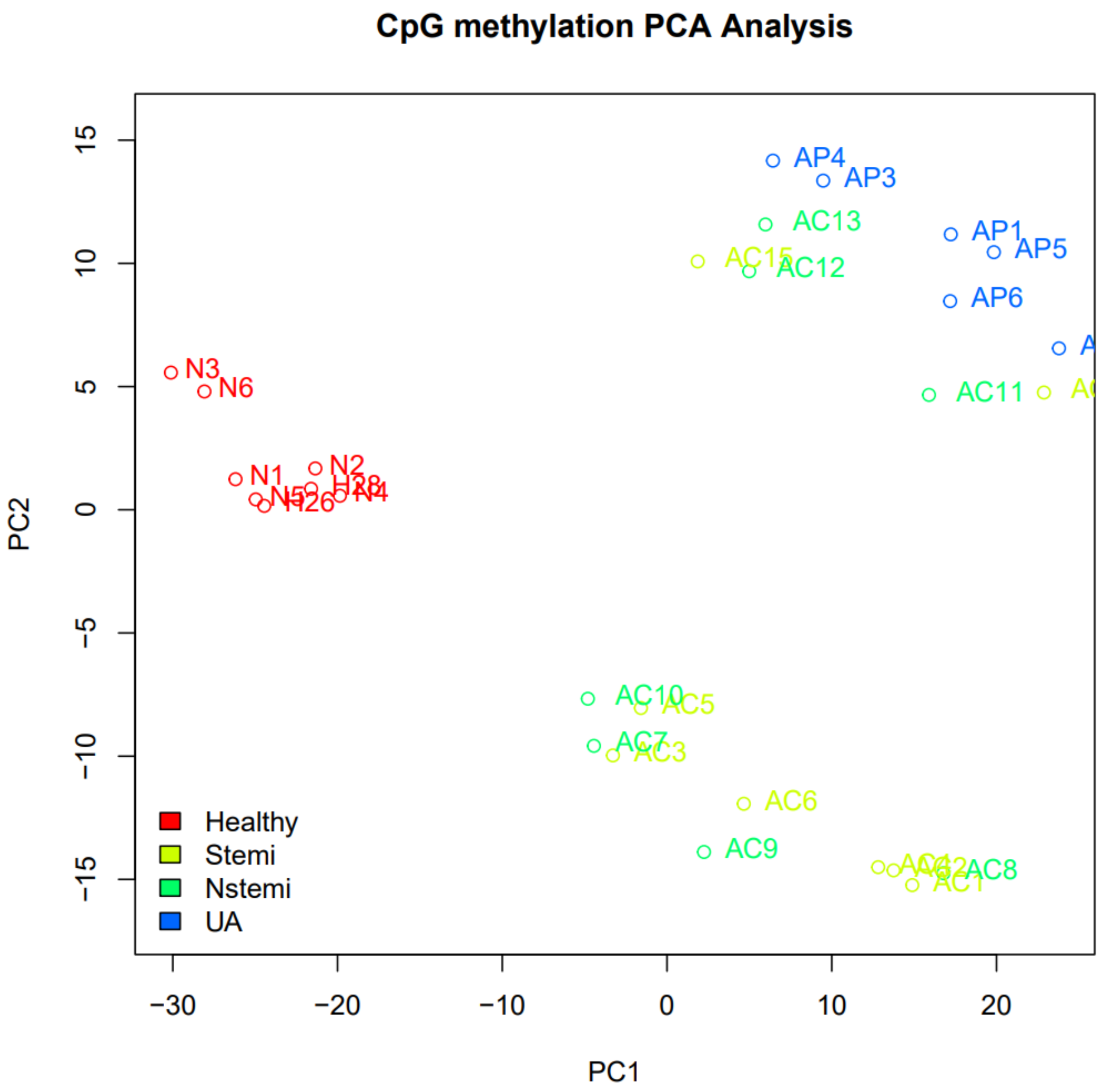

Figure S3: PCA on CpG methylation percentage using all differentially methylated regions (DMRs with at least 25% difference among 2 groups, q-value smaller than 0.01) from discovery samples.

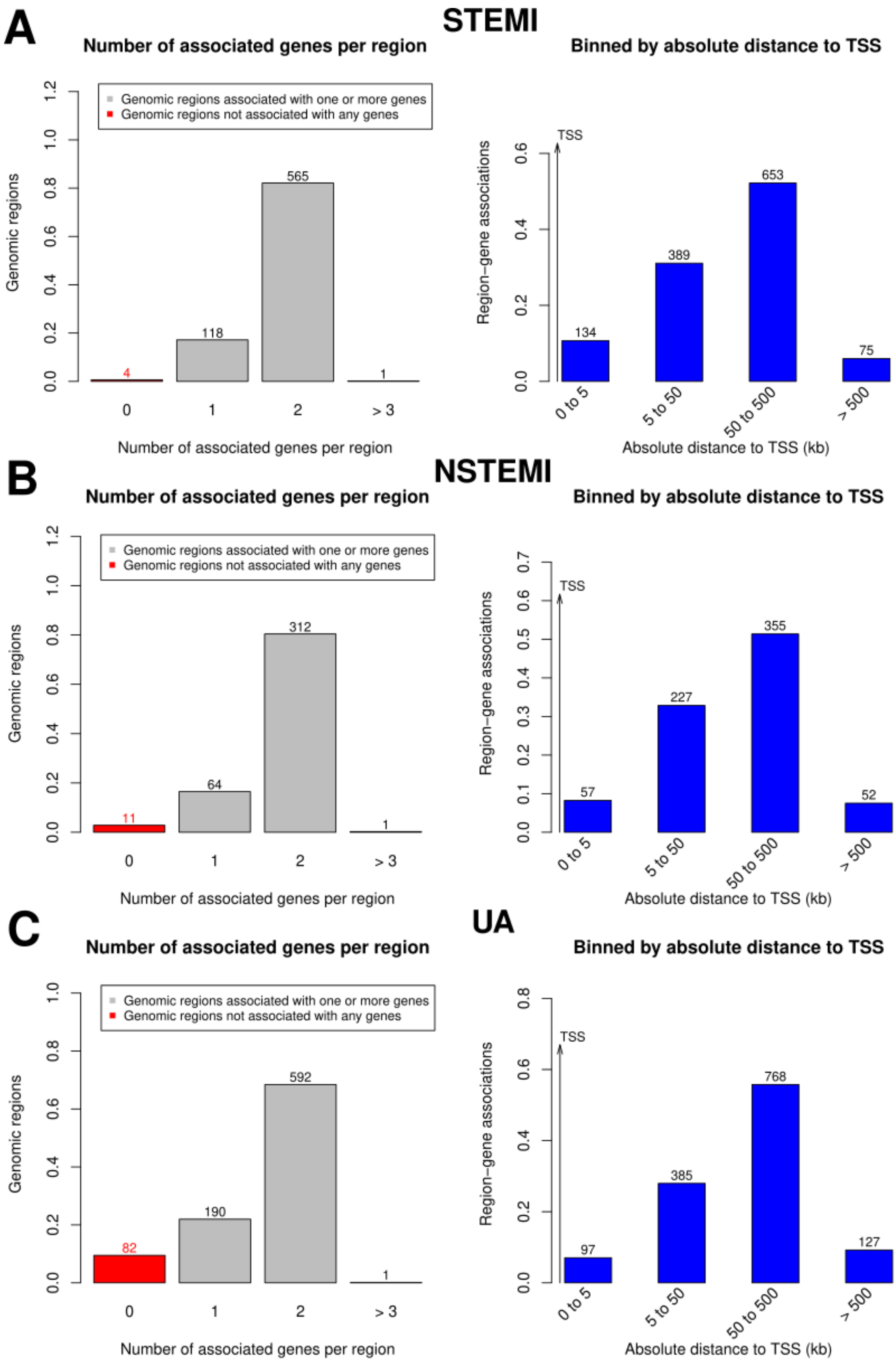

Figure S4: DMRs associated with genes and their distances to transcription start sites (TSS). The DMRs for each comparison were annotated by Genomic Regions Enrichment of Annotations Tool (GREAT) using the rGREAT package (release 3.12).

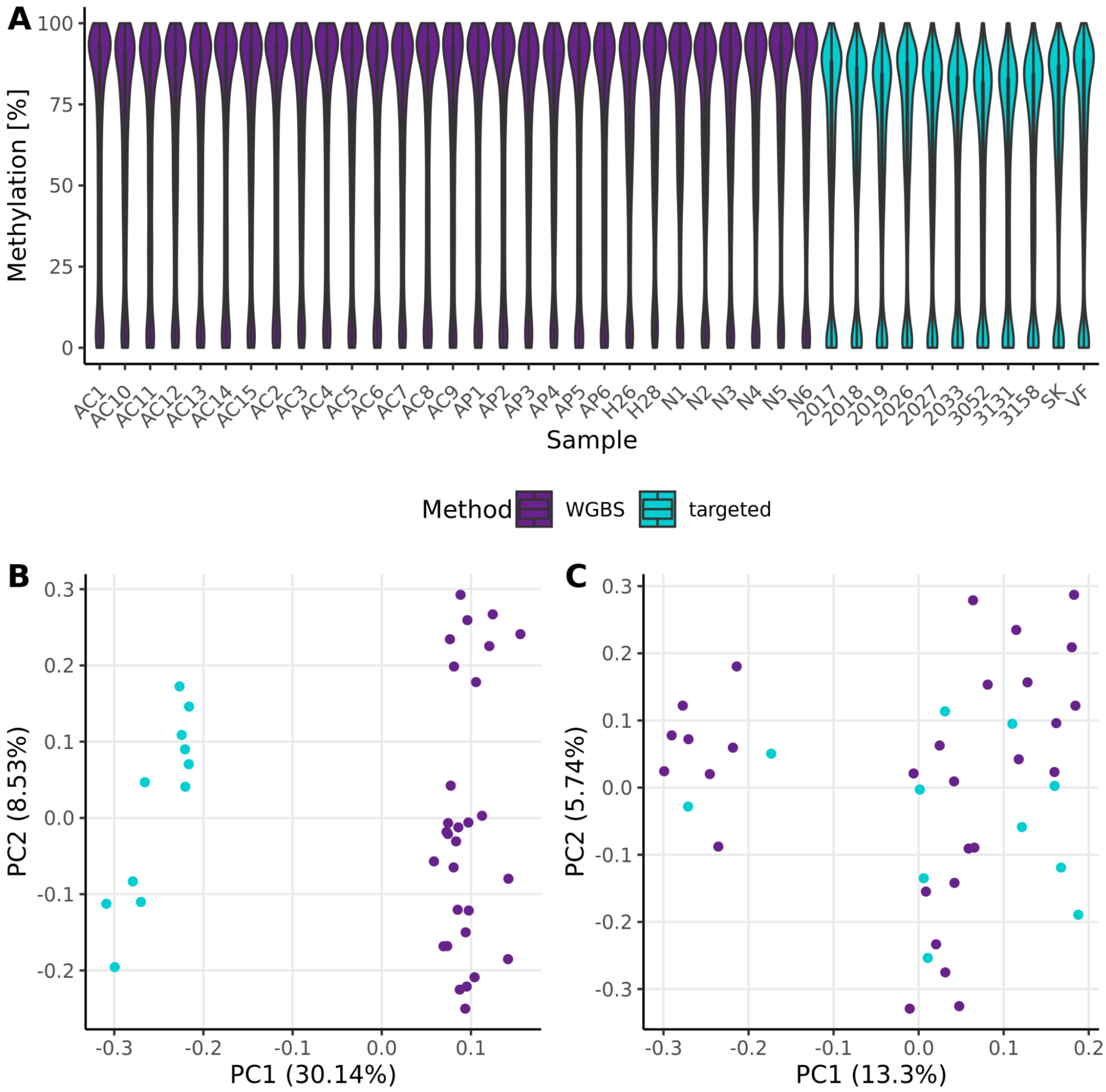

Figure S5: Removal of systematic methylation bias. A - Systematic bias between targeted sequenced samples and WGBS samples. Boxplots are depicted within the violin plots, showing hinges from 25th to 75th percentile with whiskers expanding from +/- 1.5* interquartile range of the hinges. **B - PCA on log transformed percentages of methylation from all samples on all common methylation tiles (n = 9114). C - PCA on log transformed, quantile normalized, ComBat adjusted and back transformed percentages of methylation from all samples on common methylation tiles (n = 9114).**

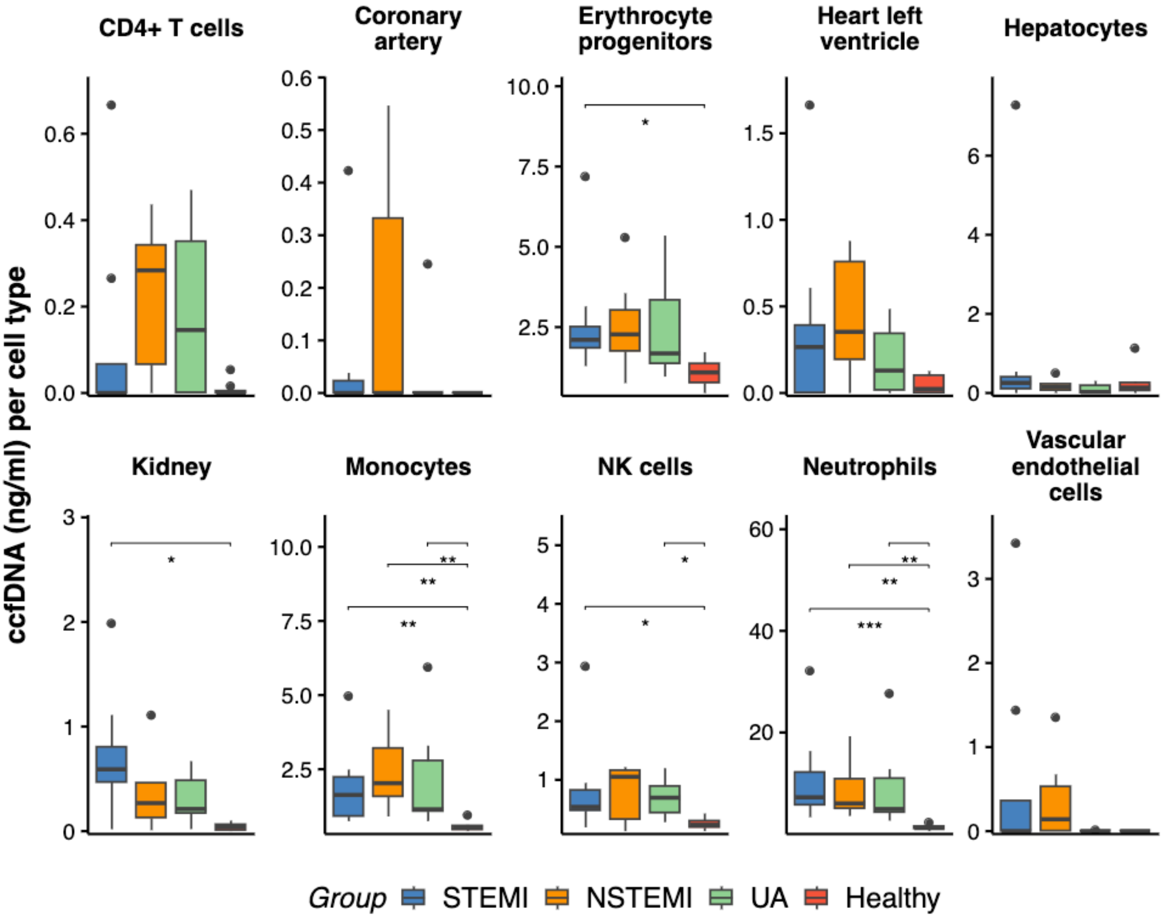

Figure S6: Predicted cell/tissue-type proportions per group normalized by the concentration of the sample's ccfDNA. The stars indicate significance levels according to Wilcoxon Test (Significance Levels *: P-Value < 0.05, **: P-Value < 0.01, ***: P-Value < 0.001)

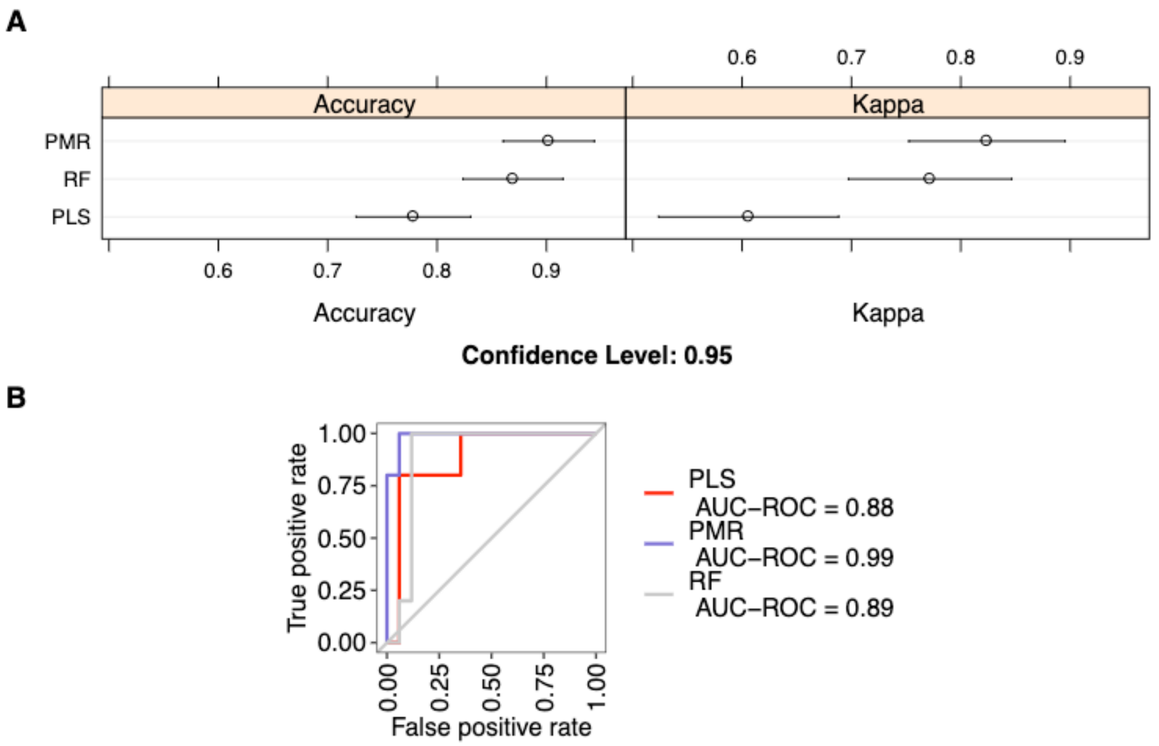

Figure S7: Machine learning approaches on ccfDNA methylation data to determine ACS type. A **-** Performance Metrics under Cross-Validation Resampling**.** B - ROC-Curve
PMR - Penalized Multinomial Regression, PLS - Partial Least Squares regression, RF - Random Forest, ROC - receiver operating characteristic, AUC-ROC - Area under the ROC curve for Cross-Validation resampling.
